# Peptide-induced mRNAs in whole blood ex vivo as a unique assay for peptide-specific T cell immunity

**DOI:** 10.1186/2051-1426-1-S1-P108

**Published:** 2013-11-07

**Authors:** Masato Mitsuhashi, Jane B Trepel, Min-Jung Lee, Sunmin Lee, Masaki Terabe, Jay A Berzofsky, Lauren V Wood

**Affiliations:** 1 Hitachi Chemical Research Center, Inc., Irvine, CA, USA; 2 Medical Oncology Branch, Center for Cancer Research, National Cancer Institute, Bethesda, MD, USA; 3 Vaccine Branch, Center for Cancer Research, National Cancer Institute, Bethesda, MD, USA

## 

Assessment of antigen-specific humoral immunity (IgG) is established and used clinically, whereas the analysis of antigen-specific cellular immunity is not applied as a routine clinical test, due to the complexity of assay procedures. Antigen specificity in cellular immunity means peptide specificity on an autologous major histocompatibility complex (MHC) molecule, which is recognized by the corresponding T cell receptors (TCR). In order to develop clinically applicable tests, we added CEF peptides into heparinized whole blood, and incubated at 37°C for 1-24 hours, then early responder-, immune function-specific mRNAs were quantified by the method we reported previously (J Immunol Methods. 363:95, 2010). The CEF peptides are 8-12 amino acids in length, with sequences derived from the human cytomegalovirus, Epstein-Barr virus, and influenza virus, and used in the stimulation of IFNγ release from CD8+ T cells in ELISPOT and other immune assays. In order to have adequate HLA-matched antigen presenting cells in the assay, we added peptide into whole blood, where both class I and II MHC molecules are available on the surface of leukocytes, which can present the peptides to autologous peripheral blood T cells. When this experiment was done in adult volunteers’ blood, we found significant induction of various cytokine and chemokine mRNAs, including IFNγ, TNFα, GMCSF, IL2, CCL4, and CXCL10, whereas the control housekeeping gene β-actin (ACTB) was not induced all the time. The degree of induction and type of induced mRNAs were varied considerably among individuals, suggesting the variation of immune functionality in each individual. The induction was very rapid and peaked around 2-4 hours. This system was successfully tested in an NCI-sponsored clinical cancer vaccine trial as an exploratory assay, and the induction of these mRNAs were confirmed after 4 hours incubation with CEF in heparinized whole blood. Moreover we also found the induction of regulatory T cell (Treg)-related mRNAs such as IL10, CD25, PDL1, and FOXP3 in a small percentage of patient samples, suggesting the possible presence of Treg cells. The induction of Th2-related mRNA (IL4), myeloid-derived suppressor cells-derived mRNA (ARG1), PD1, and CTLA4 mRNAs was not detected. In 5 patients, where blood was obtained from Day 0 to Day 168 serially, we also characterized the fluctuation of CEF-induced mRNAs. Thus, the system reported here could be practically applied to clinical settings, and by selecting mRNA species, various immune function might be assessed quantitatively.

